# The modulatory effects of facial cues and familiarity in face recognition: a behavioral and eye-tracking investigation

**DOI:** 10.3389/fpsyg.2026.1833741

**Published:** 2026-06-11

**Authors:** Gaoyuan Zhang, Yuye Yang, Hui Chen, Xurong Xie, Jin Huang, Xiaoxia Du

**Affiliations:** 1 Institute of Software, Chinese Academy of Sciences, Beijing, China; 2 China Rehabilitation Research Center, Beijing, China

**Keywords:** FAI-generated faces, eye-tracking, face recognition, familiarity, internal and external facial features, visual attention

## Abstract

**Introduction:**

Face recognition is essential for social interaction, yet the extent to which internal facial features and external facial cues contribute to identity recognition remains incompletely understood. In particular, it remains unclear how external appearance changes, such as hairstyle and hair color, influence the recognition of familiar and unfamiliar faces across perceptual and memory-based processing stages.

**Methods:**

This study examined the modulatory effects of external facial cues and face familiarity using behavioral measures and eye-tracking. Thirty-four native Chinese-speaking adults completed a modified visual paired comparison face memory task, and 31 of them also completed a face matching task. Facial stimuli included familiar Chinese celebrities and unfamiliar faces, with systematic manipulations of hairstyle and hair color. In the face matching task, participants judged whether two simultaneously presented faces depicted the same identity. In the face memory task, participants first encoded a single face and then judged recognition when the familiarized face was paired with either a different identity or the same identity with altered external cues. Eye movements were analyzed across predefined areas of interest, including internal features, internal non-feature regions, and external regions.

**Results:**

In the face matching task, external cue variations did not significantly affect accuracy but increased reaction times, particularly for unfamiliar faces. Reaction times were longer when hairstyle or hair color differed, and hair color changes showed a stronger disruptive effect for unfamiliar than familiar faces. In the face memory task, hairstyle manipulations selectively affected recognition accuracy for unfamiliar faces, whereas hair color manipulations did not show the same pattern. Eye-tracking results showed that participants consistently prioritized internal facial features across tasks. However, gaze allocation and gaze transition patterns were flexibly modulated by face familiarity and external cue variations. During memory encoding, familiar faces elicited relatively greater attention and transitions involving external regions, whereas unfamiliar faces were associated with a more internally focused scanning pattern. During recognition, external cue changes, especially hairstyle changes, increased dwell time on the target face and altered comparison-related gaze dynamics.

**Discussion:**

These findings suggest that face recognition is a dynamic process shaped by the interaction between external facial cues, familiarity, and task demands. Internal facial features remained the primary source of identity-relevant information, but external cues influenced processing efficiency, memory-based recognition, and visual exploration. Familiar faces appeared more robust to external appearance changes, whereas unfamiliar faces were more susceptible to disruptions caused by changes in external cues, particularly hairstyle. The results provide a more comprehensive account of how facial identity information is extracted and integrated across perceptual and memory-based recognition stages, and offer a foundation for future research on face recognition in populations with atypical face processing.

## 1 Introduction

Face recognition plays a critical role in social cognition, as it enables individuals to identify others and interpret socially relevant information in everyday interactions. Successful face recognition supports a wide range of social functions, including person identification and social interaction (Haxby et al., 2002; Avery et al., 2016). Impairments in face recognition may therefore have significant consequences for social functioning. For example, individuals with developmental prosopagnosia often experience persistent difficulties in recognizing facial identity, which has been associated with elevated levels of social inhibition (Avery et al., 2016). In addition, atypical face processing has been widely reported in individuals with autism spectrum disorder (ASD) and has been linked to challenges in social communication and interpersonal interaction (Gehdu et al., 2024; Liu et al., 2025). An important question in the face recognition literature concerns the types of facial information participants rely on when identifying faces. In particular, considerable attention has been devoted to understanding how information from different regions of the face contributes to identity recognition and guides visual attention during face processing (Tanaka and Simonyi, 2016).

Facial information used for identity recognition is often broadly divided into internal and external features (EF) (Jarudi and Sinha, 2003). Among these, internal facial features, including the eyes, nose, and mouth, are generally considered to play a central role in face recognition. These features provide highly diagnostic cues for distinguishing between individuals and are thought to form the core of facial identity representations (Schyns et al., 2002). Consistent with this account, previous work has suggested that internal features (IF) play an important role in supporting accurate identity recognition (Frowd et al., 2007). On the other hand, external facial features, such as hairstyle, hair color, and head or face contour, have often been more variable. Much of the early behavioral research on external facial features examined their contribution under specific conditions or for particular categories of faces (Davies et al., 1977). These works suggested that EF may be especially useful for recognizing particular faces (Sinha and Poggio, 1996, 2002). Although behavioral research has shown that EF can facilitate recognition under specific conditions or for particular types of faces, their contribution across faces more generally remains relatively underexplored. However, neuroimaging findings suggest that EF play a more important role than this limited behavioral literature might imply. Specifically, both internal and external facial features, when presented in isolation, have been found to elicit significant responses in face-selective regions such as the fusiform face area (FFA) (Andrews et al., 2015; Axelrod and Yovel, 2010). These findings indicate that EF can make meaningful contributions to the neural processing of facial identity. Taken together, the literature suggests that while IF may serve as the primary basis for face recognition, EF also play an important role, and their relative contribution may vary depending on factors such as task demands, prior experience, social relevance, and especially face familiarity (Hadders-Algra, 2022).

Previous researches have demonstrated that familiar and unfamiliar faces are processed using partially different cognitive mechanisms. Several studies have suggested that the relative importance of internal and external facial features differs depending on the type of face being processed. In particular, recognition of familiar faces appears to benefit more from information contained in internal facial features (Ellis et al., 1979). For example, when participants make identity judgments based on IF, recognition accuracy for familiar faces tends to increase compared with judgments based on external cues (Campbell, 1999; Osborne and Stevenage, 2008). In contrast, discrimination of unfamiliar faces appears to be more sensitive to external facial information. Behavioral studies have shown that recognition accuracy for some unfamiliar faces is significantly higher when EF such as hair are present than when these features are removed (Duchaine and Weidenfeld, 2003). However, this pattern has also been questioned in the literature. For instance, Ellis et al. found that when participants were provided with only internal or only external facial information, recognition accuracy for unfamiliar faces was largely comparable across the two conditions (Ellis et al., 1979). These findings suggest that the relative contributions of internal and external facial cues to face recognition remain an open question.

To better understand why familiar and unfamiliar faces may show different patterns of reliance on internal and external cues, it is useful to consider a broader theoretical distinction between holistic processing and reliance on local or individual facial cues. One possible explanation for differences between familiar and unfamiliar face recognition lies in the distinction between holistic processing and reliance on local or individual facial cues. Holistic or configural processing refers to the integration of facial features into a unified representation, with particular sensitivity to the spatial relations among features (Maurer et al., 2002; Piepers and Robbins, 2012). Previous work suggests that familiar and unfamiliar face recognition differ in important ways, with familiar face recognition being supported by more stable identity representations and unfamiliar face recognition being more vulnerable to image-based variation (Bruce and Young, 1986; Johnston and Edmonds, 2009). However, existing evidence does not clearly establish that unfamiliar face recognition depends on featural or part-based processing *per se*. Rather, it remains an open question whether unfamiliar face recognition is less strongly supported by integrated representations and therefore more susceptible to changes in salient local or external cues. This issue is particularly relevant for the current study, because changes in hairstyle and hair color may disproportionately affect recognition when observers rely more heavily on such cues.

Eye-tracking research has provided important insights into the perceptual mechanisms underlying face recognition by examining how participants allocate visual attention across different facial regions. Previous studies have shown that visual exploration during face processing typically follows a pattern focused on internal facial features, particularly the eyes, nose, and mouth (Blais et al., 2008; Peterson and Eckstein, 2012). However, gaze patterns are not fixed and may vary depending on task demands and the type of face being processed. For example, eye-tracking studies have shown that when learning to recognize unfamiliar faces, participants tend to spend more time fixating on external facial features such as hair and head contour (Henderson et al., 2005). Examining gaze allocation and gaze transitions, therefore, provides a useful approach for understanding how participants gather identity-relevant information during face recognition.

As discussed above, Previous studies report mixed findings regarding the role of external facial cues in familiar and unfamiliar face recognition. Furthermore, much of the existing research has relied on relatively simple perceptual matching paradigms (Baker et al., 2026), neglecting the crucial memory processes underlying identity storage and retrieval (Bruce and Young, 1986; Burton et al., 1990). These paradigms may not fully align with how we recognize faces in the real world, where participants must encode a face and later recognize it under varying appearance conditions. In addition, everyday external variations, such as simultaneous changes in hairstyle and hair color, are more complex than typical experimental manipulations. Yet these specific variations have rarely been systematically manipulated in previous studies. Recent advances in artificial intelligence (AI) techniques allow precise manipulation of facial representations while preserving overall visual realism (Shen et al., 2020). Importantly, previous studies have shown that participants often struggle to distinguish AI-generated faces from real photographs, suggesting that such images provide visually realistic stimuli for face perception research (Nightingale and Farid, 2022). These developments provide a powerful and efficient approach for investigating how variations in facial appearance influence face recognition.

The current study examined how changes in external facial appearance, specifically hairstyle and hair color, affect the recognition of familiar and unfamiliar faces. To address this question, we employed both matching-based and memory-based face recognition paradigms, allowing us to examine the effects of external appearance changes across different recognition contexts. Eye-tracking was also used to assess how visual attention was distributed across facial regions during recognition. More specifically, we asked whether changes in hairstyle and hair color affect face recognition, whether these effects differ between familiar and unfamiliar faces, and whether they are associated with differences in gaze allocation. We expected recognition of familiar faces to be relatively robust to changes in external appearance, whereas recognition of unfamiliar faces would be more susceptible to such changes and more strongly influenced by external facial information.

## 2 Method

### 2.1 Participant

Thirty-four native Chinese-speaking participants were recruited through advertisements and online social media groups and received monetary compensation for their participation. Participants reported normal or corrected-to-normal vision and no history of color vision deficiency. All participants were right-handed and provided written informed consent prior to participation. The study included two experimental tasks: a face matching experiment (Task 1) and a face memory experiment (Task 2). The face memory experiment included all 34 participants (18 males and 16 females), with a mean age of *M* = 25.71 years (*SD* = 4.51, range = 19–41 years). The face matching experiment included 31 participants (16 males and 15 females), with a mean age of *M* = 25.94 years (*SD* = 4.65, range = 19–41 years). Three participants (two males and one female) did not complete the face matching experiment due to scheduling constraints.

### 2.2 Materials and apparatus

Materials: All images were front-facing photographs without accessories such as glasses or necklaces. Prior to the experiment, all face images were standardized using Adobe Photoshop (Adobe Inc., San Jose, CA, USA). Images were cropped to remove background information and standardized in size so that the face, hair, and a small portion of the upper shoulders were visible. The final stimulus set included 24 identities, consisting of 12 familiar faces and 12 unfamiliar faces. Familiar faces were well-known Chinese celebrities, whereas unfamiliar faces were individuals unlikely to be recognized by the participants. To ensure gender balance, both the familiar and unfamiliar face sets included six male and six female faces. To validate the familiarity manipulation, participants rated the familiarity of each face prior to the experiment using a 5-point Likert scale (1 = completely unfamiliar, 5 = very familiar). A paired-samples *t*-test comparing familiarity ratings for familiar and unfamiliar faces confirmed a significant difference between the two categories, indicating that the familiarity manipulation was successful (*t* = 23.72, *p* < 0.001). Stimuli were displayed at the center of the screen on a uniform white background to minimize visual distractions.

The focus on hairstyle and hair color as the manipulated dimensions was theoretically motivated. Both cues constitute salient external facial information and can contribute meaningfully to face recognition (Jarudi and Sinha, 2003). In addition, hairstyle and hair color frequently vary in everyday life, making them ecologically relevant sources of appearance variation. Moreover, these manipulations can be introduced while leaving internal facial features relatively unchanged, allowing us to isolate the contribution of external appearance to recognition performance. Importantly, hairstyle and hair color may reflect different aspects of external facial information: hairstyle is closely related to the overall outline of the external face region, whereas hair color primarily alters appearance-related surface information without directly changing internal facial structure (Toseeb et al., 2012; Russell et al., 2007). Focusing on these two manipulations therefore provided a controlled way to examine whether different forms of external appearance change differentially affect the recognition of familiar and unfamiliar faces.

To manipulate hairstyle and hair color while keeping other facial features constant, additional image editing procedures were applied. Hair color was modified using Adobe Photoshop by converting the original black hair to blond. Hairstyle variations were generated using an AI-based image generation system (Doubao) to produce alternative hairstyles for the same identity while preserving all other facial characteristics. The hairstyle manipulation involved clearly noticeable categorical changes in hairstyle, such as changing short hair to long hair or straight hair to curly hair, rather than only subtle variations. To ensure that the AI-generated images did not introduce visual artifacts, a pretest was conducted prior to the main experiment. Participants viewed 12 face images and judged whether each image was AI-generated or a natural photograph. The mean accuracy was 0.54, which did not differ significantly from chance level (50%) (*t* = 1.52, *p* = 0.14). These results indicate that participants were generally unable to reliably distinguish AI-generated images from original photographs.

Apparatus: Gaze trajectories were recorded at a sampling rate of 120 Hz using a Tobii Pro X3-120 remote eye-tracking system. Stimulus presentation and gaze data acquisition were controlled using Tobii Pro Lab. Participants were seated approximately 60 cm from the monitor during the experiment. Prior to the experimental trials, a standard 5-point calibration procedure was conducted for each participant to ensure accurate gaze tracking. Calibration accuracy was assessed using a validation procedure. Recalibration was carried out whenever the maximum validation error exceeded 1.30° of visual angle. Across participants, the mean validation error was 0.45°.

### 2.3 Procedure

Participants completed two separate experiments: a face matching experiment and a face memory experiment. To minimize potential carryover effects between tasks, the two experiments were conducted in separate sessions with an interval of at least one day between them. The order of the experiments was counterbalanced across participants. Both experiments were conducted using an eye-tracking system, and participants were seated in a quiet, dimly lit environment during the experiment. The face matching experiment was designed to examine participants' reliance on external facial cues during identity judgments and the role of face familiarity in this process (Sugimura, 2013). Also, researchers employed a modified visual paired comparison (VPC) paradigm to investigate how external facial cue variations influence visual exploration and recognition during face memory(Fantz, 1964; Pascalis and de Haan, 2014). Details for the two experiments are described below.

**Figure 1:**
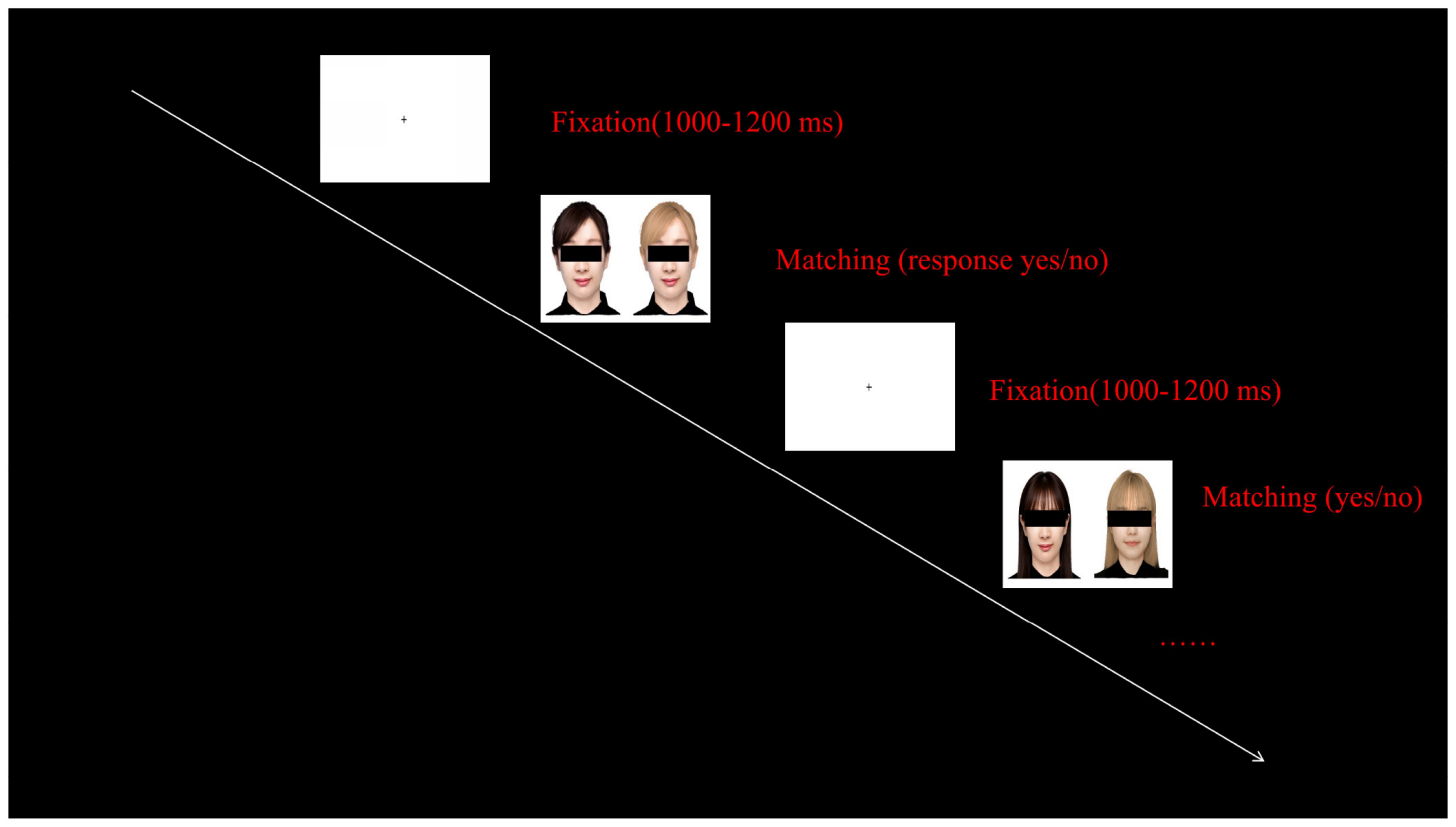
Face matching experiment protocol.

Face Matching Experiment: Each trial began with a central fixation cross presented for 1000-1200 ms, which also served as the inter-stimulus interval (ISI) between trials and directed participants' gaze to the center of the screen, following procedures commonly used in other eye-tracking experiments (Grice et al., 2005). Following the fixation period, a pair of face images was presented at the center of the screen. Participants were instructed to decide as quickly and accurately as possible whether the two faces depicted the same person or two different people and to indicate their response using designated keyboard keys (Burton et al., 2010). The stimuli remained on the screen until a response was made. The trial structure is illustrated in Figure 1.

**Figure 2:**
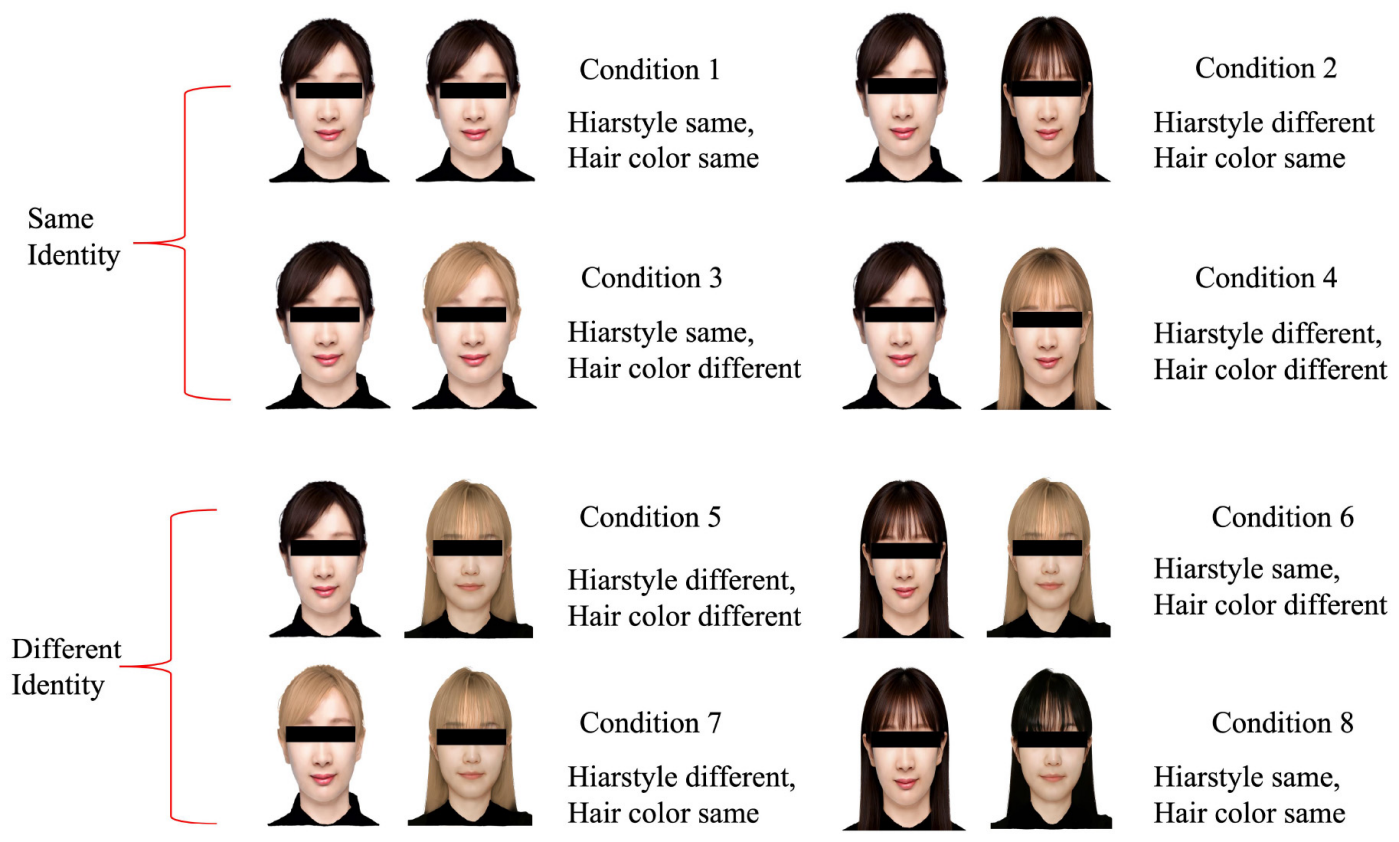
Examples of the eight experimental conditions used in the face matching experiment.

The face pairs varied in terms of identity, hairstyle, and hair color, resulting in eight experimental conditions (see Figure 2). Four trials involving the same identity, the two images could have the same hairstyle and hair color, different hairstyles but the same hair color, the same hairstyle but different hair colors, or both different hairstyles and different hair colors. For trials involving different identities, the two images could have different hairstyles and different hair colors, the same hairstyle but different hair colors, different hairstyles but the same hair color, or the same hairstyle and the same hair color. These conditions allowed us to examine how variations in hairstyle and hair color influenced identity matching judgments.

Each condition contained six trials (Chawarska and Shic, 2009). Because the same set of eight conditions was presented for both familiar and unfamiliar faces, each participant completed a total of 96 trials in the face matching task. All trial types were presented in a pseudorandom order throughout the experiment. Within each trial, the two faces were presented on the left and right sides of the screen, and their positions were counterbalanced(left or right side) across trials. The same set of conditions was applied to both familiar faces and unfamiliar faces, allowing us to examine whether familiarity modulated identity matching under different hairstyles and hair color variations.

Face Memory Experiment: Compared with the traditional VPC paradigm, several modifications were introduced in the current study. That is an additional explicit response stage was included after the recognition phase. During this stage, participants indicated how many faces presented during the recognition phase corresponded to the individual shown during the familiarization phase by pressing the designated keyboard key. Moreover, the stimulus conditions in the recognition phase were manipulated to introduce variations in external facial cues.

**Figure 3:**
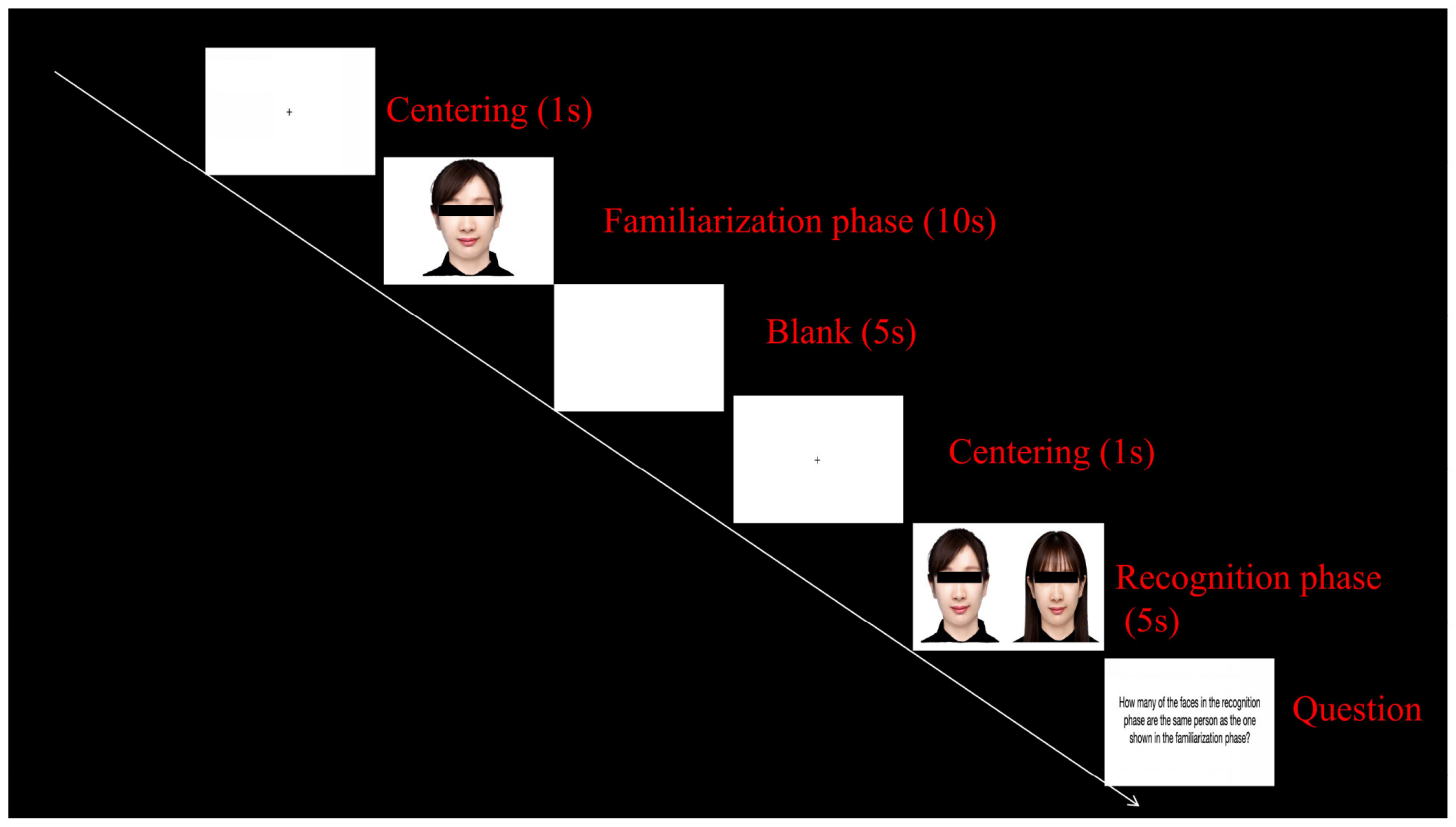
Face memory experiment protocol.

Following the general structure of VPC paradigms used in previous eye-tracking studies (Chawarska and Shic, 2009), each trial began with a central fixation cross presented for 1 s to direct participants' gaze to the center of the screen. During the familiarization phase, a single face stimulus was presented at the center of the screen for 10 s. This was followed by a blank screen for 5 s, after which a re-centering fixation cross (1 s) was presented to reorient participants' gaze. The subsequent recognition phase lasted 5 s. During this phase, the face presented during the familiarization phase was shown again and served as the old-side face, while a second face stimulus from the same stimulus set was presented on the opposite side of the screen and served as the target-side face. The whole experiment protocol can be seen in Figure 3. Depending on the experimental condition, the target-side face could depict either the same identity with modified EF or a different identity.

**Figure 4:**
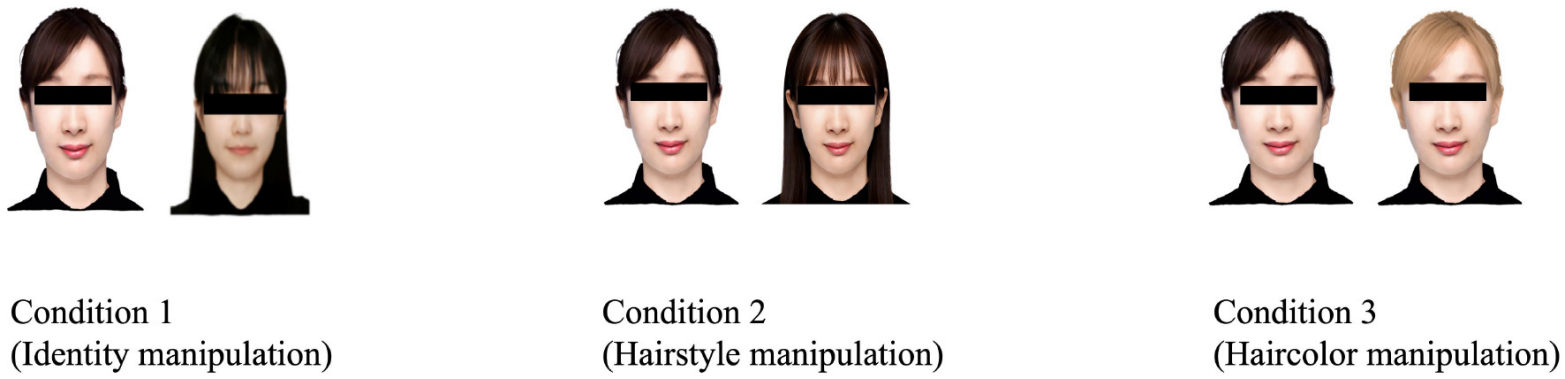
Examples of the four experimental conditions used in the recognition phase of the face memory experiment.

The experiment included both familiar faces and unfamiliar faces. Four condition types were constructed. During the recognition phase, the familiarized face could be paired with either a different identity, the same identity with a different hairstyle, or the same identity with a different hair color (see Figure 4). In addition, a further condition was included in which the familiarized face was paired with another different identity. This condition was included solely to balance response keys and was therefore excluded from the final data analyses. Similar to the face matching experiment, each condition contained six trials.Because four condition types were presented for both familiar and unfamiliar faces, each participant completed a total of 48 trials in the face memory task, of which 36 trials were included in the final analyses. All condition types were presented in a pseudorandom order, and the position of the target face in the recognition phase was counterbalanced(left or right side) across trials.

### 2.4 Data analysis

All Statistical analyses were conducted using R(4.4.1). The specific statistical analysis methods used in this study are as follows.

Behavior data analysis: For both face matching experiment and face memory experiment, accuracy data were analyzed using Firth's bias-reduced logistic regression (Suhas et al., 2023). Model estimates are reported with 95% confidence intervals (95% CI).

For reaction time (RT) analyses in the face matching task, only correct trials were included. RTs that exceeded two standard deviations from each participant's mean were excluded as outliers (Berger and Kiefer, 2021). This procedure resulted in the exclusion of 126 trials out of 2791 correct trials (4.52%). The remaining RTs were log-transformed to reduce skewness and improve normality prior to analysis. The transformed RTs were analyzed using a linear mixed-effects model(LMM). For significant effects, *post-hoc* comparisons were conducted to further examine differences between conditions.

**Figure 5:**
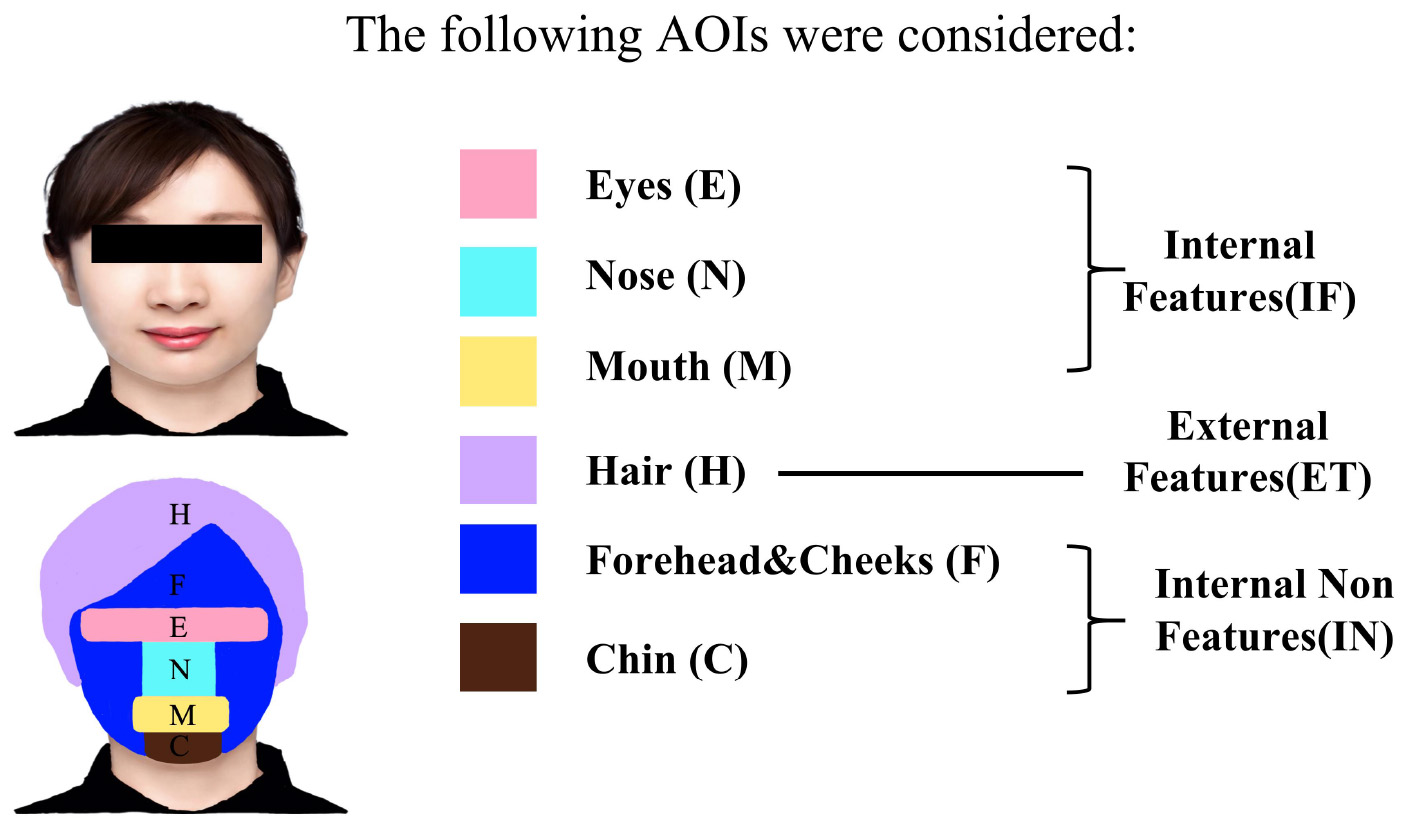
Example of area of interests (AOIs).

Eyetracking data analysis: According to previous research, to examine the spatial distribution of visual attention, the face stimuli were divided into three areas of interest (AOIs): (1) internal features(IF) (eyes, nose, and mouth), (2) internal non-features(IN) (cheeks and chin), and external features(EF) (hair) (see Figure 5) (Bennetts et al., 2025).

For the face matching task, the primary dependent variable was the total fixation duration within each AOI. Fixation durations were log-transformed prior to analysis to reduce skewness. The transformed fixation times were analyzed using linear mixed-effects models.

For the face memory task, eye-movement measures were analyzed separately for the familiarization and recognition phases. During the familiarization phase, the total fixation duration within each AOI was analyzed using a linear mixed-effects model as well. In addition, the strength of transitions between different AOIs were examined to characterize scanning patterns across facial regions.

During the recognition phase, several gaze measures were analyzed. The proportion of dwell time on the target face was analyzed using mixed-effects models. Here, the target face refers to the face that had not been presented during the familiarization phase. We also analyzed whether the first fixation landed on the target face, as well as the number of gaze switches between the previously seen face and the target face. For all models, *post-hoc* comparisons were conducted for significant main effects or interactions.

## 3 Results

### 3.1 Results of face matching experiment

Accuracy data were analyzed using Firth's bias-reduced logistic regression. Descriptive statistics for accuracy across all familiarity, hairstyle, and hair color conditions are provided in Supplementary Table S1. The analysis revealed no significant main effects or interactions, indicating that accuracy did not differ reliably across levels of Familiarity, Hairstyle, or Hair Color.

RTs were analyzed using a linear mixed-effects model with Familiarity, Hairstyle, and Hair Color as fixed effects and Participant as a random intercept. Descriptive statistics for reaction times across all experimental conditions are reported in Supplementary Table S2. The results of the model are summarized in Table 1. The analysis revealed significant main effects of Familiarity (*F* = 10.03, *p* = 0.002), Hairstyle (*F* = 24.48, *p* < 0.001), and Hair Color (*F* = 19.78, *p* < 0.001). In addition, a significant interaction between Familiarity and Hair Color was observed (*p* = 0.025), as well as a significant interaction between Hairstyle and Hair Color (*p* = 0.001). All other interaction effects were not significant.

**Table 1:** Results for the linear mixed-effects model predicting log-transformed reaction times (RTs).

Predictor	df1	df2	*F* value	*p*-value
Familiarity	1	2630.6	10.03	0.002^**^
Hairstyle	1	2628.1	24.48	< 0.001^***^
Hair color	1	2628.1	19.78	< 0.001^***^
Familiarity × Hairstyle	1	2628.0	0.43	0.512
Familiarity × Hair color	1	2628.1	5.02	0.025^*^
Hairstyle × Hair color	1	2628.1	10.38	0.001^**^
Familiarity × Hairstyle × Hair color	1	2628.1	0.15	0.692

Significance levels are indicated as follows: *p* < 0.05 (^*^), *p* < 0.01 (^**^), *p* < 0.001 (^***^).

**Figure 6:**
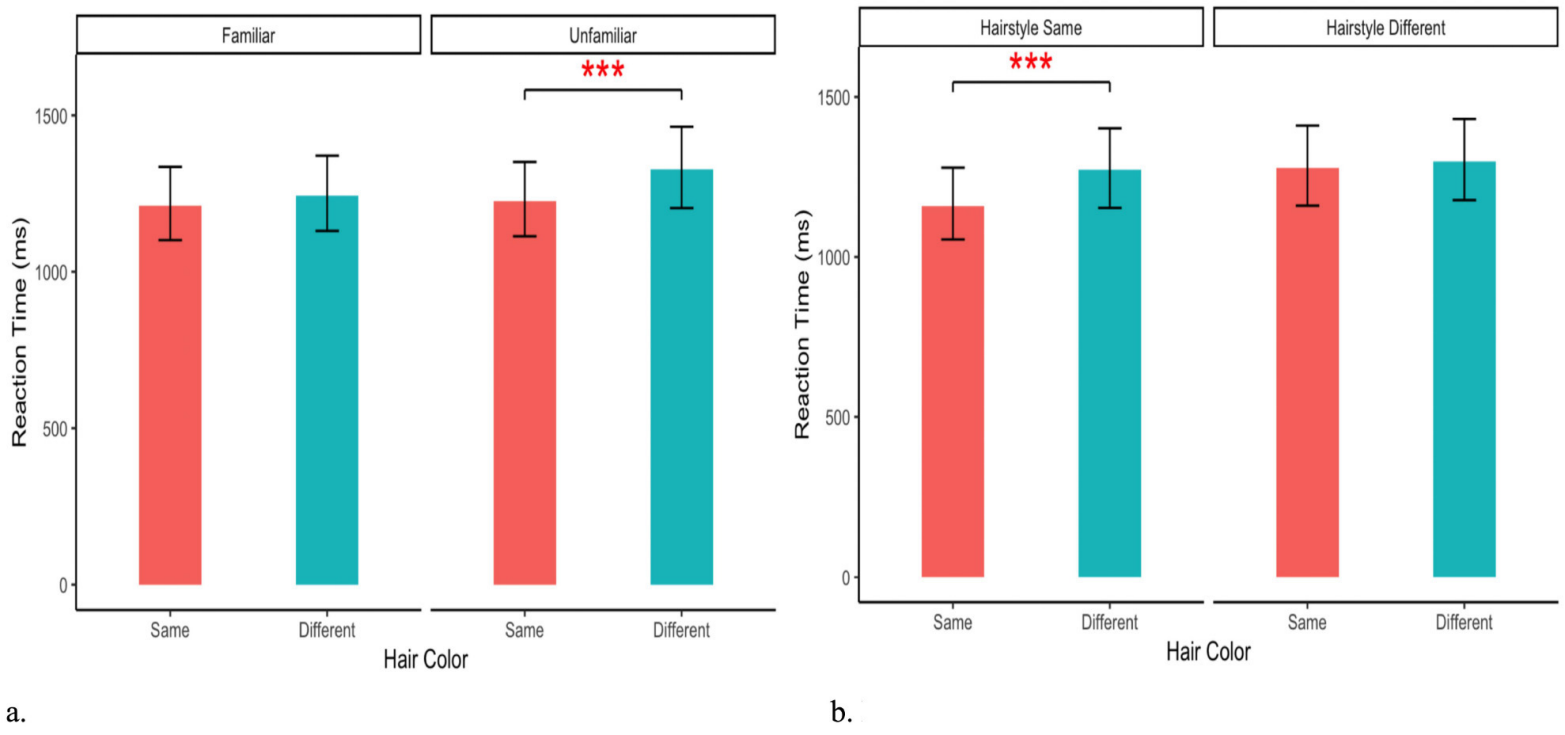
*Post-hoc* comparisons for the significant interaction effects in the face-matching task. **(a)** Interaction effect between familiarity and hairstyle. **(b)** Interaction effect between hairstyle and hair color. Significance level is indicated as follows: ^***^p < 0.001.

*Post-hoc* comparisons for the main effects are presented in Table 2. These comparisons indicated that responses were slower when hair color differed compared to when hair color was the same (*t* = −4.45, *p* < 0.001), and slower when hairstyle differed compared to when hairstyle was the same (*t* = −4.95, *p* < 0.001). In addition, responses were significantly faster for familiar faces than for unfamiliar faces (*t* = −3.17, *p* = 0.002). To further explore the significant interactions, additional analyses were conducted. These results are illustrated in Figure 6. The hair color effect was significant for unfamiliar faces (*t* = −4.64, *p* < 0.001). Similarly, the hairstyle effect was significant when hair color was the same (*t* = −5.78, *p* < 0.001). In summary, face matching accuracy did not differ reliably across conditions, whereas reaction times were influenced by familiarity, hairstyle, and hair color. Moreover, the interaction between familiarity and hair color suggests that hair color changes had a stronger disruptive effect on unfamiliar than familiar faces. Thus, in the matching task, external appearance changes primarily affected recognizing efficiency, particularly for unfamiliar faces.

**Table 2:** *Post-hoc* comparisons for the significant main effects in the face matching task.

Effect	Contrast	Estimate (β)	SE	*t* ratio	*p*-value	95% CI
Familiarity	Familiar − Unfamiliar	–0.04	0.01	–3.17	0.002^**^	[–0.06, –0.01]
Hairstyle	Same − Different	–0.06	0.01	–4.95	< 0.001^***^	[–0.08, –0.04]
Hair color	Same − Different	–0.05	0.01	–4.45	< 0.001^***^	[–0.08, –0.03]

Significance levels are indicated as follows: ^**^p < 0.01, ^***^p < 0.001.

Fixation duration during the face matching task was analyzed using a linear mixed-effects model, with AOI region and experimental conditions as fixed effects and Participant as a random intercept. Descriptive statistics for total fixation time across AOI regions and experimental conditions are provided in Supplementary Table S3. The results of the model are summarized in Table 3. The analysis revealed a significant main effect of AOI region (*F* = 150.93, *p* < 0.001). No significant main effects were observed for Hairstyle, Familiarity, or Hair Color (all *p*s >0.61). However, a significant three-way interaction between AOI region, Familiarity, and Hair Color was found (*F* = 5.64, *p* = 0.004).

**Table 3:** Linear mixed-effects model results for log-transformed AOI fixation duration in the face-matching task.

Predictor	df1	df2	*F* value	*p*-value
AOI region	2	6137.3	150.93	< 0.001^***^
Hairstyle	1	6125.8	0.01	0.912
Familiarity	1	6132.9	0.06	0.812
Hair color	1	6125.2	0.26	0.613
AOI region × Hairstyle	2	6125.8	0.10	0.370
AOI region × Familiarity	2	6131.4	0.38	0.683
Hairstyle × Familiarity	1	6125.8	1.83	0.177
AOI region × Hair color	2	6126.0	0.55	0.578
Familiarity × Hair color	1	6126.3	2.49	0.115
AOI region × Hairstyle × Familiarity	2	6125.9	2.87	0.057
AOI region × Hair color × Familiarity	2	6125.9	5.64	0.004^**^

Significance levels are indicated as follows: ^**^p < 0.01, ^***^p < 0.001.

*Post-hoc* comparisons for the main effect of AOI region are presented in Table 4. These comparisons revealed that fixation durations differed significantly across all three regions. Participants spent more time fixating on IF than IN regions (*z* = −13.27, *p* < 0.001), and ET (*z* = −12.95, *p* < 0.001). In addition, fixation durations were significantly longer for IN regions compared to ET (*z* = −3.82, *p* < 0.001). To further decompose the significant interaction among AOI region, Familiarity, and Hair Color, we examined whether fixation patterns across AOI regions differed between familiar and unfamiliar faces separately within each hair color condition. This difference was significant when hair color differed(F = 3.29, p = 0.037), but did not reach significance when hair color was the same(F = 2.61, p = 0.07). Pairwise comparisons were then conducted to compare fixation durations across AOI regions separately for each familiarity and hair color condition.

**Table 4:** *Post-hoc* comparisons for the main effect of AOI region.

Contrast	Estimate (β)	SE	*z* ratio	*p*-value	95% CI
ET − IN	–0.15	0.04	–3.82	< 0.001^***^	[–0.24, –0.06]
ET − IF	–0.46	0.04	–12.95	< 0.001^***^	[–0.54, –0.38]
IN − IF	–0.31	0.02	–13.27	< 0.001^***^	[–0.36, –0.25]

AOI regions: IF, internal feature; IN, internal non-feature; ET, external region. Significance levels is indicated as follows: ^***^p < 0.001.

**Figure 7:**
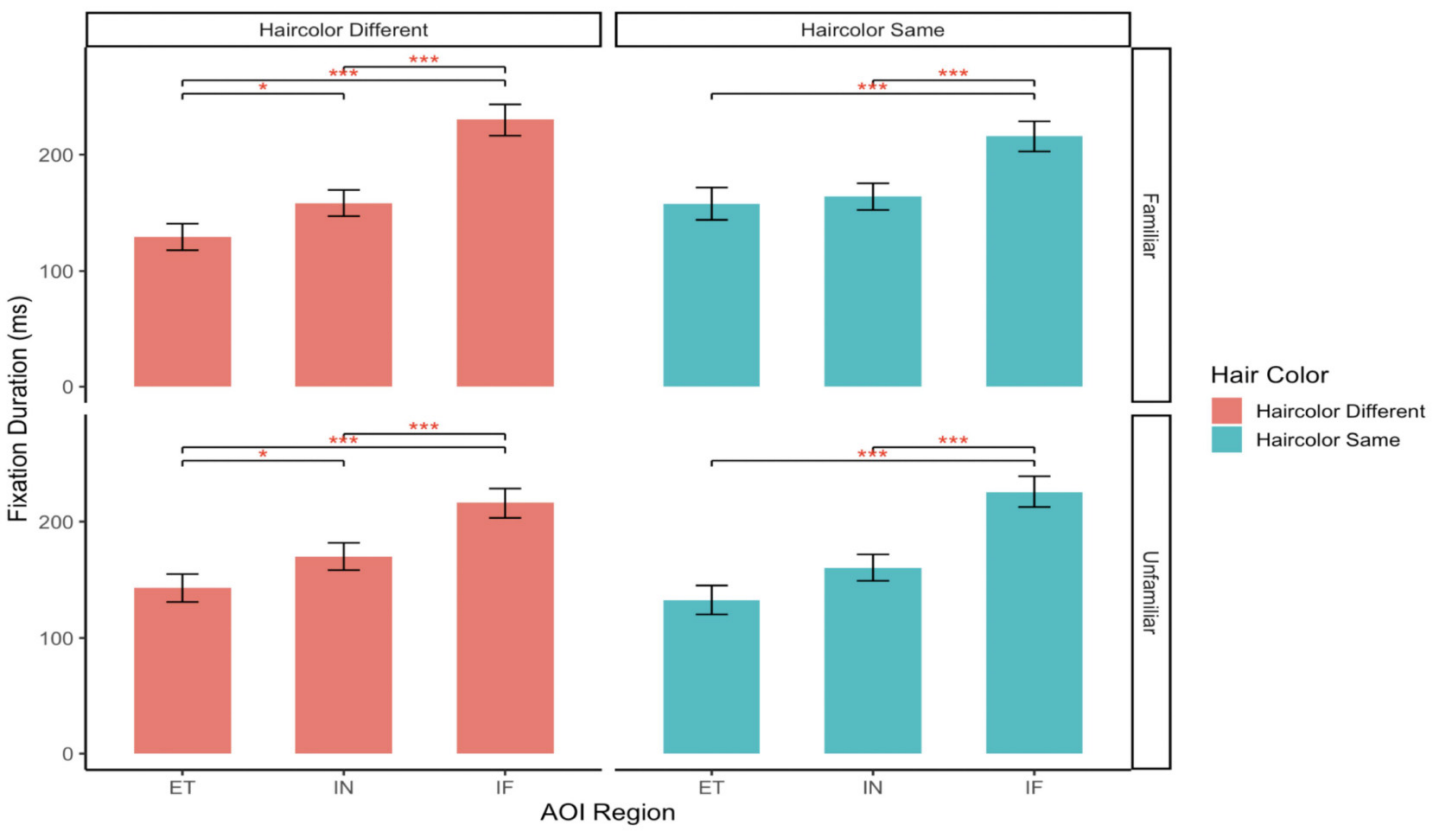
Fixation duration across AOI regions as a function of familiarity and hair color. Significance levels are indicated as follows: ^*^p < 0.05, ^***^p < 0.001.

As illustrated in Figure 7, pairwise comparisons showed that fixation durations on IF were consistently higher than those on IN and ET regions across all familiarity and hair color conditions. When hair color differed, fixation durations on ET regions were significantly lower than those on IN regions for both familiar faces(z = −2.56, p = 0.028), and unfamiliar faces(z = −2.38, p = 0.045). In contrast, when hair color was the same, the difference between ET and IN regions was not significant for either familiar or unfamiliar faces. In addition, fixation durations on IF were significantly longer than those on both ET and IN regions across all four familiarity and hair color combinations (all ps < s 0.001).

Additional comparisons were conducted to examine familiarity differences within each hair color and AOI region condition. When hair color differed, familiar faces showed significantly longer fixation durations than unfamiliar faces in the IF region(z = 2.36, p = 0.018), whereas no significant familiarity differences were observed in the ET or IN regions. When hair color was the same, no significant familiarity differences were observed in any AOI region. Overall, these results indicate that participants consistently prioritized internal facial features during face matching, while the relative distribution of fixation time across AOI regions varied as a function of both familiarity and hair color condition. In particular, when hair color differed, ET received less attention than IN, and familiarity-related differences were mainly reflected in fixation duration on IF.

### 3.2 Results of face memory experiment

Accuracy in the face memory task was analyzed using Firth's bias-reduced logistic regression, with Familiarity and Condition as predictors. Descriptive statistics for memory accuracy across familiarity and condition are reported in Supplementary Table S4. The results of the model are summarized in Table 5. A significant interaction between Familiarity and Condition was observed for the hairstyle condition (*z* = −2.15, *p* = 0.031).

**Table 5:** Firth's bias-reduced logistic regression model results for the face memory task.

Predictor	Estimate	SE	*z* value	*p*-value
Familiarity	2.40	1.48	1.62	0.106
Condition (hairstyle)	–0.32	0.57	–0.56	0.575
Condition (hair color)	–0.57	0.55	–1.04	0.300
Familiarity × Condition (hairstyle)	–3.34	1.55	–2.15	0.031^*^
Familiarity × Condition (hair color)	–1.66	1.59	–1.04	0.299

Condition (Hairstyle) refers to the hairstyle manipulation condition; Condition (Hair color) refers to the hair color manipulation condition. Significance level is indicated as follows: ^*^p < 0.05.

**Figure 8:**
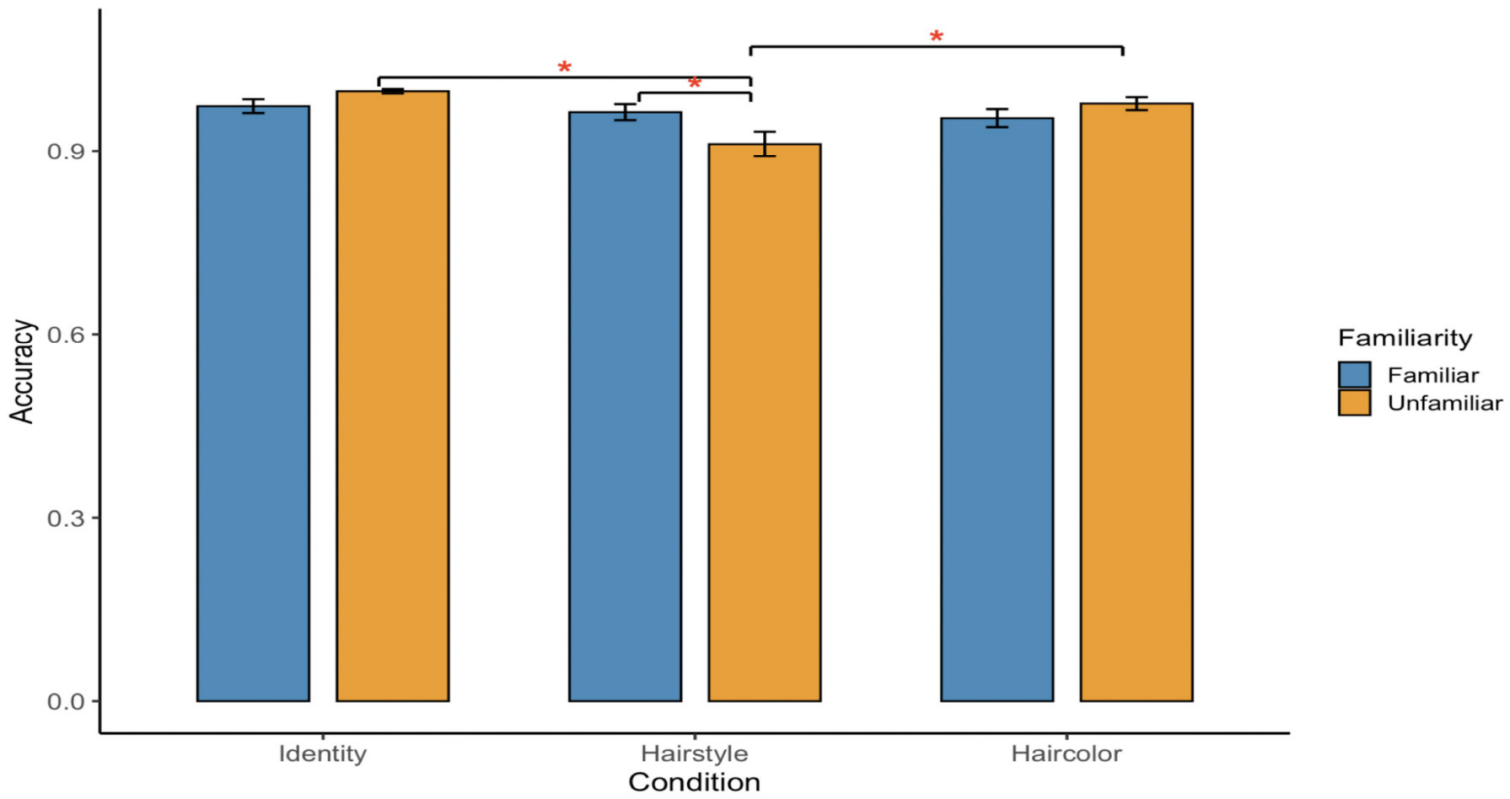
AOI regions: IF, internal feature; IN, internal non-feature; ET, external region. Post-hoc comparisons for the significant interaction effects of accuracy in the face memory task. Conditions: Identity = Condition 1 (identity manipulation condition); Hairstyle = Condition 2 (hairstyle manipulation condition); Haircolor = Condition 1 (haircolor manipulation condition). Significance level is indicated as follows: ^*^p < 0.05.

To further examine this interaction, pairwise comparisons were conducted within each familiarity level. For familiar faces, no significant differences were found between the three conditions. In contrast, for unfamiliar faces, accuracy differed significantly between the identity manipulation and hairstyle manipulation conditions (*z* = 2.54, *p* = 0.033) and between the hairstyle manipulation and hair color manipulation conditions (*z* = −2.66, *p* = 0.023). No significant difference was observed between the identity manipulation and hair color manipulation conditions. Additional comparisons examining familiarity effects within each condition showed that accuracy was significantly higher for unfamiliar faces than familiar faces in the hairstyle condition (*z* = 2.10, *p* = 0.036). No significant familiarity differences were observed in the identity or hair color conditions. These results indicate that the effect of familiarity depended on whether hairstyle changes were involved, as illustrated in Figure 8. In summary, hairstyle changes selectively altered recognition performance in the memory task, particularly for unfamiliar faces, whereas hair color changes did not produce the same pattern. This suggests that hairstyle manipulations played a more critical role than hair color manipulations in modulating memory-based face recognition.

Fixation duration during the familiarization phase of the face memory task was analyzed using a linear mixed-effects model, with AOI region and Familiarity as fixed effects and Participant as a random intercept. Descriptive statistics for AOI fixation time during the familiarization phase are provided in Supplementary Table S5. The results of the model are summarized in Table 6. The analysis revealed significant main effects of AOI region (*F* = 847.32, *p* < 0.001) and Familiarity (*F* = 5.47, *p* = 0.019), as well as a significant interaction effect between AOI region and Familiarity (*F* = 15.47, *p* < 0.001).

**Table 6:** Linear mixed-effects model results for fixation duration during the familiarization phase.

Predictor	df1	df2	*F* value	*p*-value
AOI region	2	6583.9	847.32	< 0.001^***^
Familiarity	1	6328.4	5.47	0.019^*^
AOI region × Familiarity	2	6583.9	15.47	< 0.001^***^

Significance levels are indicated as follows: ^*^p < 0.05, ^**^p < 0.001.

**Figure 9:**
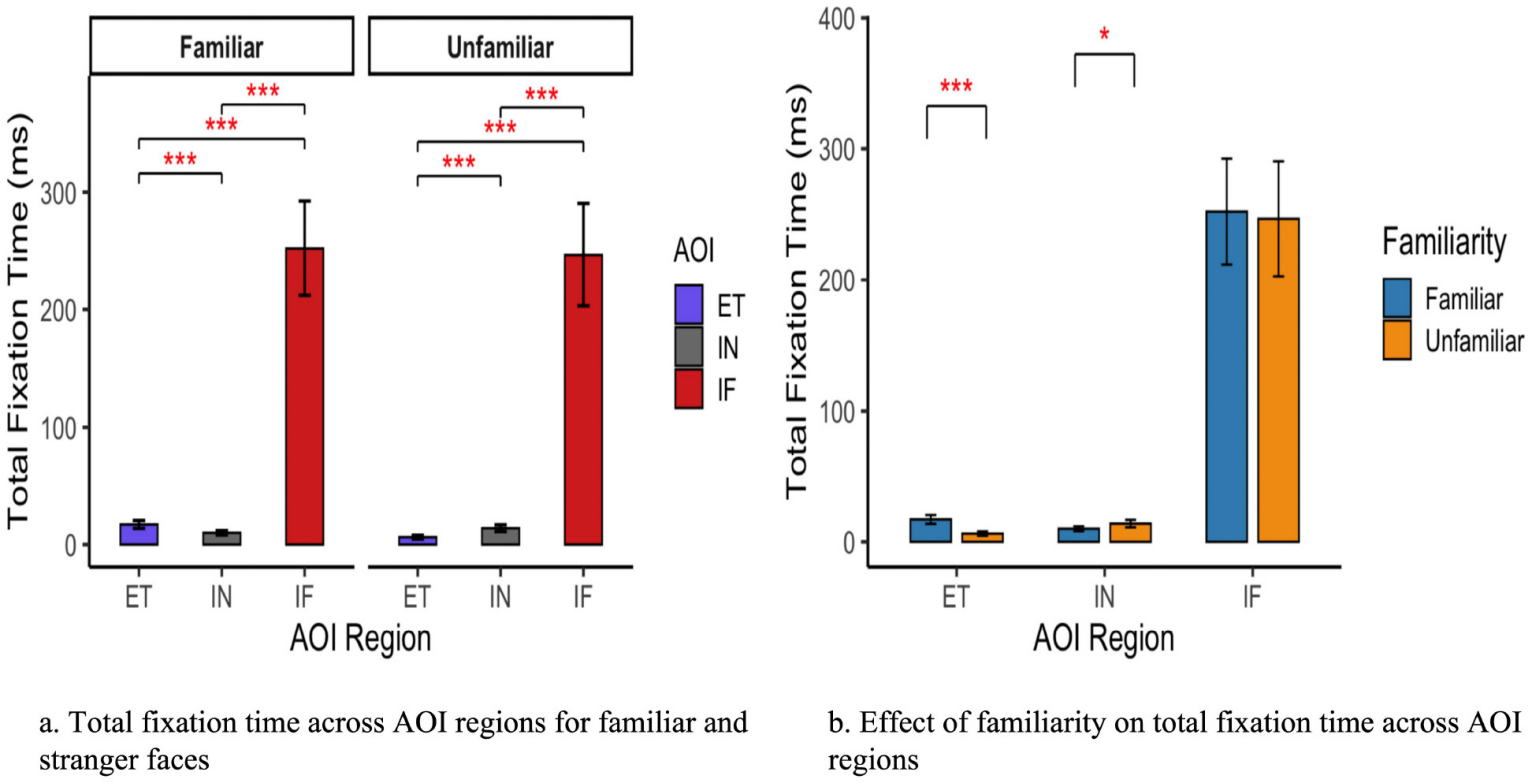
Fixation duration across AOI regions as a function of face familiarity during the familiarization phase. **(a)** Total fixation time across AOI regions for familiar and stranger faces. **(b)** Effect of familiarity on total fixation time across AOI regions. Significance levels are indicated as follows: *p < 0.05, ***p < 0.001.

*Post-hoc* comparisons (Table 7) showed that fixation durations were significantly longer for IF than for both IN and ET (all *p*s < 0.001). In addition, fixation durations were overall longer for familiar than unfamiliar faces (*z* = 2.34, *p* = 0.019). Simple-effects analyses (Figure 9) further revealed that for familiar faces, fixation duration was longer for ET than for IN (*z* = 3.92, *p* < 0.001), whereas IF received the longest fixation durations (all *p*s < 0.001). In contrast, for unfamiliar faces, fixation duration was longer for IN than for ET (*z* = −4.00, *p* < 0.001), while IF again received the longest fixation durations (all *p*s < 0.001). Additional comparisons showed that familiar faces received longer fixation durations than unfamiliar faces in ET (*z* = 4.99, *p* < 0.001), whereas unfamiliar faces received longer fixation durations in IN (*z* = −2.28, *p* = 0.022). No familiarity difference was observed for IF (*p* = 0.842). Overall, participants allocated the greatest amount of visual attention to IF during familiarization phase. At the same time, familiarity modulated the relative distribution of attention between ET and IN regions.

**Table 7:** *Post-hoc* comparisons for the main effect of AOI regions and familiarity.

Effect	Contrast	Estimate (β)	SE	*z* ratio	*p*-value	95% CI
AOI region	ET − IN	–0.11	0.11	–1.00	0.578	[–0.37, 0.15]
	ET − IF	–3.08	0.10	–29.95	< 0.001^***^	[–3.33, –2.84]
	IN − IF	–2.97	0.08	–36.21	< 0.001^***^	[–3.17, –2.78]
Familiarity	Familiar − Unfamiliar	0.21	0.09	2.34	0.019^*^	[0.03, 0.38]

Significance levels are indicated as follows: ^*^p < 0.05, ^***^p < 0.001.

To examine gaze transitions during the familiarization phase, the proportion of transitions among AOIs was analyzed using a linear mixed-effects model with Transition Type (IF↔ET, IF↔IN, IN↔ET) and Familiarity as fixed effects and Participant as a random intercept. Descriptive statistics for AOI transition proportions are reported in Supplementary Table S6. The results of the model are summarized in Table 8. The analysis revealed a significant main effect of transition type (*F* = 1304.00, *p* < 0.001), indicating that transition frequencies differed across categories (see Table 9). Meanwhile, a significant interaction effect between Transition Type and Familiarity was observed (*F* = 21.12, *p* < 0.001).

**Table 8:** Linear mixed-effects model results for AOI transition proportions during the familiarization phase.

Predictor	df1	df2	*F* value	*p*-value
AOI region transitions	2	3,273	1304.00	< 0.001^***^
Familiarity	1	3,273	0.12	0.733
AOI region transitions × Familiarity	2	3,273	21.12	< 0.001^***^

Significance level is indicated as follows: ^***^p < 0.001.

**Table 9:** *Post-hoc* comparisons for the main effect of transition type.

Effect	Contrast	Estimate (β)	SE	*z* ratio	*p*-value	95% CI
AOI region transitions	IF↔ET − IF↔IN	–1.66	0.04	–37.59	< 0.001^***^	[–1.77, –1.56]
	IF↔ET − IN↔ET	0.49	0.04	11.14	< 0.001^***^	[0.39, 0.60]
	IF↔IN − IF↔ET	2.16	0.04	48.73	< 0.001^***^	[2.05, 2.26]

Significance level is indicated as follows: ^***^p < 0.001.

**Figure 10:**
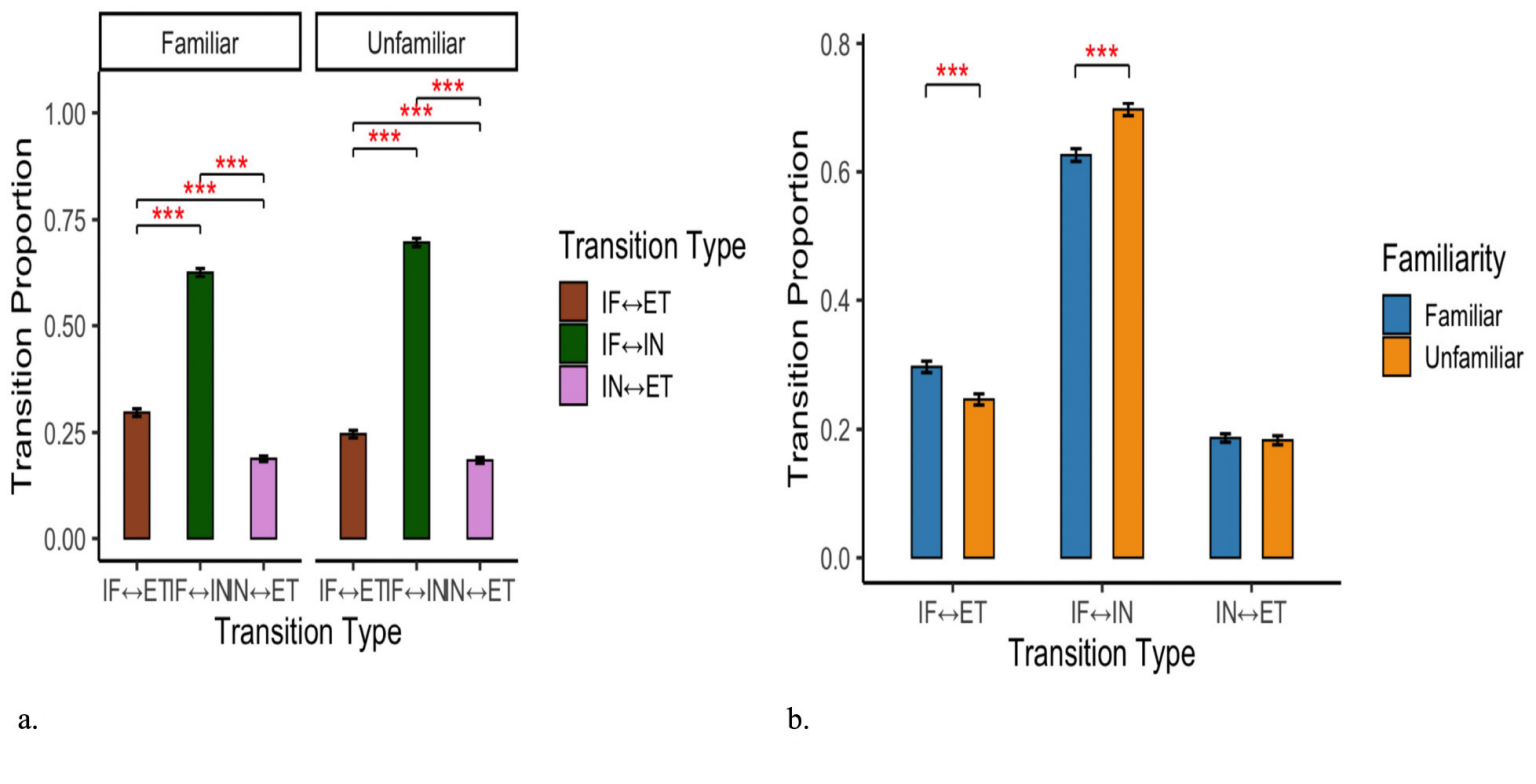
AOI transition proportions as a function of transition type and face familiarity during the familiarization phase. **(a)** Transition proportions across transition types for familiar and strange faces. **(b)** Effect of familiarity on transition proportions across transition types. Significance levels is indicated as follows: ^***^p < 0.001.

*Post hoc* comparisons showed that transitions between IF and IN occurred significantly more frequently than transitions between IF and ET and IN and ET (both *p*s < 0.001), and IF↔ET transitions occurred more frequently than IN↔ET transitions (*p* < 0.001). As shown in Figure 10a, gaze transitions during the familiarization phase were therefore primarily concentrated between IF and IN regions across both familiar and unfamiliar faces, followed by transitions between IF and ET, whereas transitions between IN and ET occurred least frequently.

Simple-effects analyses further showed that familiarity modulated these transition patterns. As shown in Figure 10b, IF↔ET transitions were more frequent for familiar than unfamiliar faces (*z* = 4.08, *p* < 0.001), whereas IF↔IN transitions were more frequent for unfamiliar than familiar faces (*z* = −5.06, *p* < 0.001). No familiarity difference was observed for transitions between IN and ET. Together, these results indicate that familiarity selectively modulated transitions involving external facial regions, suggesting different scanning strategies for familiar and unfamiliar faces during memory familiarization.

To investigate visual attention during the recognition phase, the proportion of dwell time on the target face was analyzed using a linear mixed-effects model with Familiarity and Condition as fixed effects and Participant as a random intercept. Descriptive statistics for target dwell proportion across familiarity and condition are provided in Supplementary Table S7. The results of the model are summarized in Table 10. The analysis showed a significant main effect of Condition (*F* = 24.87, *p* < 0.001).

**Table 10:** Linear mixed-effects model results for target dwell proportion during the recognition phase.

Predictor	df1	df2	*F* value	*p*-value
Familiarity	1	884.50	0.17	0.683
Condition	2	269.95	24.87	< 0.001^***^
Familiarity × Condition	2	869.80	1.02	0.362

Significance level is indicated as follows: ^***^p < 0.001.

*Post-hoc* pairwise comparisons (Tukey-adjusted) for the main effect of Condition are presented in Table 11. These analyses showed that the proportion of dwell time on the target face was significantly higher in the hairstyle condition than in the identity condition (*t* = −7.05, *p* < 0.001) and in the hair color condition than in the identity condition (*t* = −3.59, *p* = 0.001). In addition, the hairstyle condition showed a significantly higher target dwell proportion than the hair color condition (*t* = 3.44, *p* = 0.002). In summary, participants spent a greater proportion of time viewing the target face in the hairstyle and hair color conditions than in the identity condition, with the hairstyle condition showing the strongest effect. These results suggest that changes in external appearance influenced how visual attention was allocated during recognition phase.

**Table 11:** *Post-hoc* comparisons for the main effect of Condition (target dwell proportion).

Effect	Contrast	Estimate (β)	SE	*t* ratio	*p*-value	95% CI
Condition	Identity − Hairstyle	–0.16	0.02	–7.05	< 0.001^***^	[–0.22, –0.11]
	Identity − Hair color	-0.08	0.02	-3.59	0.001^**^	[–0.14, –0.03]
	Hairstyle − Hair color	0.08	0.02	3.44	< 0.002^**^	[0.03, 0.13]

Significance levels are indicated as follows: ^**^p < 0.01, ^***^p < 0.001.

To examine early attentional orienting during the recognition phase, the probability that the first fixation landed on the target face was analyzed using a linear mixed-effects model with Familiarity and Condition as fixed effects and Participant as a random intercept. Descriptive statistics for first fixation on the target face are reported in Supplementary Table S8. The analysis revealed no significant main effects of Familiarity or Condition, and no significant interaction effect (all *p*s >0.34). Thus, early attentional orienting during recognition was not reliably influenced by familiarity or condition.

To further understand gaze dynamics during the recognition phase, we analyzed the count of gaze switches between the old face and the target face. Descriptive statistics for switch counts across familiarity and condition are provided in Supplementary Table S9. Switch counts were analyzed using a linear mixed-effects model with Familiarity and Condition as fixed effects and Participant as a random intercept. The results of the model are summarized in Table 12. The analysis revealed a significant main effect of Condition (*F* = 10.57, *p* < 0.001).

**Table 12:** Linear mixed-effects model results for switch counts between the old side and the target side during the recognition phase.

Predictor	df1	df2	*F*-value	*p*-value
Familiarity	1	887.64	0.01	0.935
Condition	2	864.83	10.57	< 0.001^***^
Familiarity × Condition	2	864.81	2.71	0.067

Significance level is indicated as follows: ^***^p < 0.001.

*Post-hoc* pairwise comparisons (see Table 13) for the main effect of Condition indicated that switch counts were significantly lower in the hair color condition than in the identity condition (*t* = 2.76, *p* = 0.016) and lower than in the hairstyle condition (*t* = 4.57, *p* < 0.001). The difference between the identity and hairstyle conditions was not significant. Overall, participants made fewer gaze switches in the hair color condition than in the other conditions, suggesting reduced comparison between the old and target faces when only hair color was manipulated.

**Table 13:** *Post-hoc* comparisons for the main effect of Condition (switch counts).

Effect	Contrast	Estimate (β)	SE	*t* ratio	*p*-value	95% CI
Condition	Identity − Hairstyle	–0.33	0.18	–1.86	0.151	[–0.75, 0.09]
	Identity − Haircolor	0.49	0.18	2.76	0.016^*^	[0.07, 0.91]
	Hairstyle − Haircolor	0.83	0.18	4.57	< 0.001^**^	[0.40, 1.25]

Significance levels are indicated as follows: ^*^p < 0.05, ^**^p < 0.01.

## 4 Discussion

The current study investigated how variations in external facial cues and face familiarity jointly influence identity processing by examining both behavioral performance and eye-movement patterns across the face matching task and the face memory task. Overall, the findings suggest that although variations in external facial cues (specifically hairstyle and hair color) had no impact on judgment accuracy, they significantly modulated processing efficiency and visual exploration patterns across both matching and memory tasks.Importantly, these effects were modulated by face familiarity, suggesting an interaction between external cue variations and familiarity in shaping both behavioral performance and eye-movement patterns.

### 4.1 Effects of external facial cues and face familiarity on behavioral performance of face identity

#### 4.1.1 Face matching performance

Behavioral results from the face matching task showed that variations in external facial cues did not affect the accuracy, but influenced the efficiency of identity judgments. Specifically, reaction times were longer when hairstyle or hair color differed between faces, whereas accuracy remained at ceiling and did not differ significantly across conditions. Importantly, these effects were modulated by face familiarity, with changes in hair color exerting a stronger influence on unfamiliar faces. In addition, the effect of hairstyle depended on hair color, indicating that different types of external cues interacted in shaping identity matching performance. Together, this pattern suggests that external cue variations increased the time required for identity judgments, particularly for unfamiliar faces, without affecting participants' ability to correctly determine identity. This pattern is broadly consistent with previous research demonstrating that external facial features can influence face recognition, particularly at the level of processing efficiency. Previous neuroimaging evidence further shows that external facial features elicit significant responses in face-selective regions such as the FFA when presented in isolation (Andrews et al., 2010), suggesting that such cues contribute meaningfully to face processing. Notably, these effects appear to be especially pronounced for unfamiliar faces. Consistent with the present findings, behavioral studies have reported a relative advantage of EF in unfamiliar face recognition (Duchaine and Weidenfeld, 2003; Frowd et al., 2007). For example, Duchaine and Weidenfeld (2003) found that participants could recognize unfamiliar faces at near-normal levels even when internal facial features were removed, indicating a substantial contribution of external information under these conditions.

One possible explanation for this pattern is that face recognition in typical adults relies on structural encoding of facial identity rather than simple pictorial matching (Young et al., 1985). Within this framework, participants form relatively stable mental representations that integrate information across both internal and external facial features. At the same time, face processing is shaped by prior perceptual experience and social interactions, reflecting top-down influences that guide efficient extraction of identity-relevant information (Hadders-Algra, 2022). When such representations are well established, as in familiar faces, top-down mechanisms can support stable recognition despite variations in appearance. In contrast, when identity representations are less robust, as in unfamiliar faces, processing relies more heavily on bottom-up perceptual input, making it more susceptible to variations in external cues. As a result, EF may increase the effort required for feature integration, particularly during early stages of perceptual processing.

The present findings further extend prior work by demonstrating that not only global EF, but also more subtle and ecologically valid variations, such as changes in hairstyle and hair color, are sufficient to modulate the efficiency of identity judgments. Moreover, the effects of different types of external cues were not uniform. The interaction between familiarity and hair color suggests that hair color exerted a stronger influence on identity judgments for unfamiliar faces than hairstyle. One possible explanation is that hair color represents a visually salient cue that can be processed rapidly at early stages of perception, consistent with models of visual attention in which color serves as a low-level feature guiding bottom-up processing (Itti and Koch, 2001). Such bottom-up influences may play a greater role when top-down identity representations are less robust, thereby amplifying the effect of hair color in unfamiliar face processing. In addition, the interaction between hairstyle and hair color indicates that external cues do not operate independently but interact in shaping perceptual processing, consistent with evidence that face recognition involves the integration of multiple facial cues rather than isolated feature processing (Tanaka and Simonyi, 2016; Young et al., 1985).

#### 4.1.2 Face memory performance

A complementary pattern emerged in the face memory task, further clarifying the role of external facial cues across different stages of face processing. In contrast to the face matching task, where external cue manipulations primarily affected processing efficiency, the memory task revealed that recognition accuracy depended on both familiarity and the type of external manipulation. Specifically, hairstyle changes selectively affected recognition accuracy for unfamiliar faces, whereas no such effects were observed for familiar faces. This suggests that external cues not only influence perceptual comparison but also affect the stability of identity representations in memory (Andrews et al., 2015). Importantly, this dissociation between hairstyle and hair color provides further insight into the mechanisms underlying memory-based recognition. The effect of hairstyle, but not hair color, suggests that different external cues exert distinct influences on encoding and retrieval processes, particularly when identity representations are less robust (Baddeley, 2020). One possible explanation is that hairstyle changes may alter the global contour and configural structure of the face, thereby making the encoding and subsequent retrieval of identity information more difficult. Face recognition is often thought to rely on configural processing, including sensitivity to the spatial relationships among facial features and overall facial structure (Mondloch et al., 2002). Manipulations of external contour, such as hairstyle changes, may therefore interfere with the formation or use of stable holistic representations (Maurer et al., 2002). Consistent with this possibility, previous work has suggested that altering hair information can disrupt face recognition performance, potentially by influencing holistic processing (Toseeb et al., 2012). However, the this did not directly test this mechanism, and this interpretation should therefore be treated with caution.

Taken together, the findings from both tasks suggest a unified account in which external facial cues influence face processing at multiple stages, from early perceptual comparison to memory-based recognition. Crucially, their impact depends on both the type of external cue and the robustness of identity representations. When representations are stable, as in familiar faces, recognition is relatively resistant to external variations. In contrast, when representations are weaker, as in unfamiliar faces, both perceptual processing and memory encoding become more susceptible to external cue manipulations.

### 4.2 Effects of external facial cues and face familiarity on visual pattern during face processing

#### 4.2.1 Eye movements in face matching

Eye tracking results provide further insight into the processing mechanisms underlying the face matching experiment. Participants predominantly fixated internal facial features (IF), with significantly longer fixation duration compared to both IN and external regions (ET). In addition, fixation durations on IN regions were longer than those on ET regions. This finding is consistent with previous eye-tracking research showing that face processing is typically characterized by a visual sampling pattern centered on internal facial features, particularly the eyes, nose, and mouth (Peterson and Eckstein, 2012). This pattern suggests that participants preferentially allocated attention to internal facial regions that are widely considered to contain diagnostic information for identity processing (Blais et al., 2008). In turn, this may help explain the behavioral findings, in which external cue manipulations influenced reaction times but did not affect accuracy. Because identity-relevant information was primarily extracted from internal facial features, variations in external cues increased processing time without substantially interfering with identity judgments, thereby preserving overall accuracy.

Although the overall pattern of gaze allocation was stable, it was modulated by the interaction between familiarity and hair color. Specifically, when hair color differed, fixation durations on ET regions were significantly lower than those on IN regions for both familiar and unfamiliar faces. In contrast, when hair color was the same, this difference between ET and IN regions was no longer significant. This suggests that variations in hair color selectively influenced how attention was distributed across facial regions. One possible explanation for this pattern is that when hair color differs, the external region provides a highly salient but potentially unreliable cue for identity processing. While color is known to capture attention rapidly through bottom-up mechanisms (Itti and Koch, 2001), such information may be quickly evaluated as less diagnostic for identity, particularly because hair represents a highly variable feature across encounters (Latif and Moulson, 2022). As a result, participants may downweight the contribution of external regions and reallocate attention toward internal facial areas that provide more stable and reliable identity information. This dynamic interplay between bottom-up salience and top-down weighting may account for the reduced fixation on external regions when hair color varies (Navalpakkam and Itti, 2005).

At a broader level, these findings suggest that visual exploration during face matching is not entirely fixed, but dynamically adjusted depending on both perceptual input and prior knowledge. While internal facial features remain the primary focus of attention, external cue variations and familiarity jointly shape how attention is allocated across the face. This supports the view that face recognition of typical adults does not rely on a rigid reliance on a single set of facial features.

#### 4.2.2 Eye movements in face memory

Unlike the matching task, which relies on online perceptual comparison, the familiarization phase of the memory task requires participants to encode identity representations that will later be retrieved (Baddeley, 2020). As for familiarization phase, similar to the findings from the face matching task, participants predominantly fixated IF during the familiarization phase. This pattern might suggest that identity encoding relies primarily on information located within internal facial regions and closely parallels the visual strategies observed during identity comparison. It is also in line with previous eye-tracking research showing that face processing is characterized by preferential sampling of IF, such as the eyes, nose, and mouth (Blais et al., 2008; Peterson and Eckstein, 2012; Young et al., 1985), which are critical for forming stable mental representations. Notably, this preference was robust across familiarity conditions, indicating that IF provide a stable foundation for identity encoding for both familiar and unfamiliar faces.

On the other hand, familiarity modulated how attention was distributed across facial regions. For familiar faces, participants showed increased fixation on ET relative to IN, whereas for unfamiliar faces, attention was more concentrated within internal regions, with greater fixation on IN than ET. This pattern differs from previous findings (Henderson et al., 2005), which reported increased attention to external regions during the encoding of unfamiliar faces. In contrast, the present results revealed a stronger preference for internal regions, suggesting that attention remained primarily focused on identity-relevant information. One possible explanation for this discrepancy is that the contribution of EF during encoding depends on task demands (Logan et al., 2017). In this study, participants were required to encode facial identity for a subsequent recognition task in which they needed to determine how many faces corresponded to the same individual. Such task requirements may have encouraged participants to prioritize more stable and diagnostic features to maximize accuracy. For unfamiliar faces, whose identity representations are not yet established, participants may therefore rely more heavily on internal facial features, such as the eyes, nose, and mouth, which provide structurally stable information for identity encoding. In contrast, for familiar faces, internal facial information may already be well-represented in memory and can be readily retrieved without extensive encoding. As a result, participants may adopt a broader sampling strategy that includes external regions, leading to relatively increased fixation on ET compared to IN. This suggests that visual encoding strategies are flexibly adjusted based on both prior familiarity and task demands.

A similar pattern was observed in gaze transitions, providing further insight into the dynamic aspects of visual processing. Consistent with previous research suggesting that face perception involves coordinated sampling within internal regions (Butcher et al., 2025), transitions were primarily concentrated between IF and IN across conditions. However, familiarity modulated transitions involving external regions: familiar faces elicited more frequent transitions between IF and ET regions, whereas unfamiliar faces showed increased transitions between IF and IN regions. This difference extends prior work by demonstrating that familiarity not only affects where attention is allocated, but also how information is integrated across facial regions. One possible explanation is that, because of the task demands we discussed above, familiar faces are supported by more robust and abstract identity representations, allowing participants to adopt a broader and more flexible sampling strategy that incorporates both internal and external cues (Logan et al., 2017). Therefore, the observed differences in gaze transitions likely reflect an interaction between the robustness of identity representations and task-specific processing goals. While unfamiliar faces elicit a more constrained, internally focused strategy driven by the need to construct identity representations, familiar faces permit a broader sampling strategy in which both internal and external information can be integrated more efficiently.

During the recognition phase of the face memory task, eye-tracking results revealed that visual attention was systematically modulated by external cue manipulations. Specifically, the proportion of dwell time on the target face showed a significant main effect of condition, with the highest dwell time observed in the hairstyle condition, followed by the hair color condition, and the lowest in the identity condition. One possible explanation for this pattern is that manipulations of external facial cues, particularly hairstyle, introduced a mismatch between the encoded representation from the familiarization phase and the currently presented target face. When the same identity was presented with altered EF, internal facial features signaled identity consistency, whereas EF suggested a discrepancy (Gustafsson et al., 2022). This conflict may have increased cognitive load and required additional processing to resolve, leading participants to allocate more attention to the target face (Jin et al., 2025). In contrast, when the faces differed in identity, discrepancies in IF alone may have been sufficient to support rapid discrimination, reducing the need for prolonged inspection.

A similar pattern was observed in gaze switching behavior. Switch counts between the previously seen face and the target face also showed a significant main effect of condition, with more frequent switching in the hairstyle and identity conditions compared to the hair color condition, and no significant difference between the hairstyle and identity conditions. This might suggest that both identity differences and more substantial external manipulations (e.g., hairstyle changes) required more intensive comparison processes, reflected in increased gaze shifts. Compared with traditional VPC paradigms, which primarily index novelty preference without requiring explicit identity judgments, the present paradigm involved an additional identity comparison process between the previously encoded face and the target face (Chawarska and Shic, 2009). Within this comparison framework, gaze switching can be interpreted as reflecting iterative evaluation between competing identity representations. In contrast, the reduced switching observed in the hair color condition may indicate that salient perceptual cues facilitated more efficient decisions, reducing the need for repeated comparisons (Itti and Koch, 2001).

In summary, these findings suggest external facial cues influence not only whether attention is allocated to a given face, but also how visual comparison unfolds over time, reflecting the interaction between perceptual input and stored mental representations.

### 4.3 Limitation and future work

Several limitations of the current study should be acknowledged. First, the manipulation of external facial cues was restricted to variations in hairstyle and hair color. Although these features represent common changes in everyday social interactions, external facial information encompasses a broader range of elements, such as head contour and accessories. Therefore, the present findings may capture only a subset of the ways in which external appearance influences face recognition. In addition, the participant sample consisted solely of typical adults, whose face recognition abilities are generally supported by stable identity representations and efficient visual exploration strategies. As a result, the extent to which these findings generalize to populations with atypical face processing remains uncertain, as such individuals may rely on different cues or visual strategies during face recognition.

Future research may extend this investigation to neurodiverse populations who encounter significant challenges in face recognition, such as individuals with developmental prosopagnosia and ASD. Although previous literature has documented distinct gaze patterns and neural responses in these groups (Golarai et al., 2006; Tanaka and Simonyi, 2016), the specific interplay between face familiarity and the utilization of external facial cues remains under-explored in these contexts. By applying the current experimental framework to these populations, future studies may clarify whether their recognition difficulties stem from an inability to integrate internal and EF or a reduced sensitivity to familiarity, thereby offering a clearer basis for understanding the mechanisms underlying face recognition difficulties in these populations.

In addition, although behavioral pretests in the current confirmed the realism of AI-generated stimuli, the current findings rely primarily on behavioral outcomes. The next step for future research may be to systematically employ multimodal approaches to investigate whether there are subtle differences in how AI-generated vs. real faces are processed. Such investigations will be essential to ensure that the advantages of AI-driven stimulus generation can be safely extended to larger-scale studies and more diverse clinical populations without introducing confounding processing biases.

## 5 Conclusion

This study systematically investigated the role of external facial cues and face familiarity across both face matching and face memory-based recognition using a combination of behavioral measures and eye-tracking techniques. By incorporating multiple experimental conditions involving variations in familiarity, hairstyle, and haircolor, the results demonstrate that external facial cues reliably influence face matching processing, primarily by modulating matching efficiency rather than accuracy.

Across tasks, the findings of behavior performance reveal that the impact of external cues is not uniform but interacts with face familiarity and task demands. In the face matching task, external cue manipulations increased reaction times, particularly for unfamiliar faces, while maintaining high accuracy. In the face memory task, hairstyle manipulations selectively impaired memory-based recognition performance for unfamiliar faces, indicating that different external cues exert distinct effects on memory encoding and retrieval processes.Eye-tracking analyses further showed that face processing is characterized by a stable prioritization of internal facial features, while also exhibiting flexible adjustments in gaze allocation and transition patterns in response to manipulations of external facial cues and face familiarity. These findings indicate that, although IF remain the primary focus of attention, visual exploration strategies are dynamically modulated by both external appearance and prior experience during identity encoding and comparison.

Together, these findings highlight that face recognition is a dynamic process shaped by the interaction between external appearance and familiarity. The results provide a more comprehensive account of how identity information is extracted and integrated across perceptual and memory-based stages, offering a theoretical foundation for future research on face recognition of both typical populations and atypical populations.

## Data Availability

The datasets presented in this article are not readily available because email to corresponding author. Requests to access the datasets should be directed to HC, chenhui@iscas.ac.cn.
