# Supplementary material for: The modulatory effects of facial cues and familiarity in face recognition: a behavioral and eye-tracking investigation
Source: Front Psychol. 2026 Jun 11;17:1833741. doi: 10.3389/fpsyg.2026.1833741 (PMC13294385; doi:10.3389/fpsyg.2026.1833741)
Supplement: Supplementary file 1 [file Data_Sheet_1.PDF]

Supplementary Material

1 SUPPLEMENTARY TABLES

Table S1. Supplementary Table S1. Descriptive statistics for face-matching accuracy across conditions

Familiarity	Hairstyle	Hair color	Mean	SD
Familiar	Same	Same	0.99	0.07
Familiar	Same	Different	0.99	0.07
Familiar	Different	Same	0.99	0.12
Familiar	Different	Different	0.99	0.12
Unfamiliar	Same	Same	0.99	0.10
Unfamiliar	Same	Different	0.98	0.13
Unfamiliar	Different	Same	0.98	0.15
Unfamiliar	Different	Different	0.98	0.15

Note. Means and standard deviations are reported as proportions.

Table S2. Supplementary Table S2. Descriptive statistics for face-matching reaction times across conditions

Familiarity	Hairstyle	Hair color	Mean (ms)	SD (ms)
Familiar	Same	Same	1230.58	330.19
Familiar	Same	Different	1355.47	538.17
Familiar	Different	Same	1372.74	391.58
Familiar	Different	Different	1360.84	427.73
Unfamiliar	Same	Same	1331.42	521.14
Unfamiliar	Same	Different	1487.72	526.55
Unfamiliar	Different	Same	1424.81	478.11
Unfamiliar	Different	Different	1513.36	657.49

Note. Reaction times are reported in milliseconds (ms).

Table S3. Supplementary Table S3. Descriptive statistics for total fixation time across conditions in the face-matching task

AOI region	Familiarity	Hairstyle	Hair color	Mean (ms)	SD (ms)
ET	Familiar	Different	Different	151.39	71.82
ET	Familiar	Different	Same	164.42	66.03
ET	Familiar	Same	Different	189.07	140.30
ET	Familiar	Same	Same	210.45	93.61
ET	Unfamiliar	Different	Different	189.87	119.18
ET	Unfamiliar	Different	Same	163.87	81.77
ET	Unfamiliar	Same	Different	167.30	71.26
ET	Unfamiliar	Same	Same	170.57	81.76
IN	Familiar	Different	Different	193.14	71.82
IN	Familiar	Different	Same	214.86	111.83
IN	Familiar	Same	Different	177.11	83.01
IN	Familiar	Same	Same	175.81	58.95
IN	Unfamiliar	Different	Different	217.99	125.33
IN	Unfamiliar	Different	Same	177.46	85.40
IN	Unfamiliar	Same	Different	174.06	82.46
IN	Unfamiliar	Same	Same	205.94	118.81
IF	Familiar	Different	Different	296.10	120.59
IF	Familiar	Different	Same	284.16	121.70
IF	Familiar	Same	Different	288.82	118.52
IF	Familiar	Same	Same	265.99	91.31
IF	Unfamiliar	Different	Different	267.95	117.12
IF	Unfamiliar	Different	Same	306.55	137.34
IF	Unfamiliar	Same	Different	300.18	142.52
IF	Unfamiliar	Same	Same	303.87	137.02

Note. ET = External; IN = Internal Nonfeature; IF = Internal Feature. Fixation times are reported in milliseconds (ms).

Table S4. Supplementary Table S4. Descriptive statistics for memory accuracy across familiarity and conditions in the face-memory task

Familiarity	Condition	Mean	SD
Familiar	Identity	0.98	0.09
Familiar	Hairstyle	0.97	0.09
Familiar	Hair color	0.96	0.12
Unfamiliar	Identity	1.00	0.00
Unfamiliar	Hairstyle	0.92	0.14
Unfamiliar	Hair color	0.98	0.05

Note. Means and standard deviations are reported as proportions.

Table S5. Supplementary Table S5. Descriptive statistics for AOI fixation time during the familiarization phase of the face-memory task

AOI region	Familiarity	Mean (ms)	SD (ms)
ET	Familiar	264.87	250.17
ET	Unfamiliar	155.33	194.26
IN	Familiar	219.39	152.79
IN	Unfamiliar	261.12	211.62
IF	Familiar	1280.52	627.13
IF	Unfamiliar	1068.08	699.70

Note. ET = External; IN = Internal Nonfeature; IF = Internal Feature. Fixation times are reported in milliseconds (ms).

Table S6. Supplementary Table S6. Descriptive statistics for AOI transition proportions across familiarity conditions during the familiarization phase of the face-memory task

AOI region	Familiarity	Mean	SD
IF↔ET	Familiar	0.27	0.16
IF↔ET	UnFamiliar	0.20	0.12
IF↔IN	Familiar	0.65	0.17
IF↔IN	UnFamiliar	0.74	0.16
IN↔ET	Familiar	0.08	0.05
IN↔ET	UnFamiliar	0.07	0.06

Note. ET = External; IN = Internal Nonfeature; IF = Internal Feature. Means and standard deviations are reported as proportions.

Table S7. Supplementary Table S7. Descriptive statistics for target dwell proportion across familiarity and condition during the recognition phase of the face-memory task

Familiarity	Condition	Mean	SD
Familiar	Identity	0.45	0.12
Familiar	Hairstyle	0.64	0.10
Familiar	Hair color	0.57	0.10
Unfamiliar	Identity	0.49	0.15
Unfamiliar	Hairstyle	0.62	0.14
Unfamiliar	Hair color	0.50	0.20

Note. Means and standard deviations are reported as proportions.

Table S8. Supplementary Table S8. Descriptive statistics for first fixation on target across familiarity and condition duing the recognition phase of the face-memory task

Familiarity	Condition	Mean	SD
Familiar	Identity	0.54	0.18
Familiar	Hairstyle	0.56	0.19
Familiar	Hair color	0.53	0.21
Unfamiliar	Identity	0.54	0.22
Unfamiliar	Hairstyle	0.62	0.27
Unfamiliar	Hair color	0.46	0.31

Note. Means and standard deviations are reported as proportions.

Table S9. Supplementary Table S9. Descriptive statistics for switch count across familiarity and condition in the recognition phase of the memory task

Familiarity	Conditions	Mean	SD
Familiar	Identity	3.68	1.66
Familiar	Hairstyle	3.56	1.57
Familiar	Hair color	2.91	1.33
Unfamiliar	Identity	3.20	1.58
Unfamiliar	Hairstyle	3.72	1.88
Unfamiliar	Hair color	2.79	1.65

Note. Means and standard deviations are reported as counts.
